# Electroconvulsive Shock, but Not Transcranial Magnetic Stimulation, Transiently Elevates Cell Proliferation in the Adult Mouse Hippocampus

**DOI:** 10.3390/cells10082090

**Published:** 2021-08-14

**Authors:** Tian Rui Zhang, Evelyn Guilherme, Aydan Kesici, Alyssa M. Ash, Fidel Vila-Rodriguez, Jason S. Snyder

**Affiliations:** 1 Department of Psychology, University of British Columbia, Vancouver, BC V6T 1Z4, Canada; tianrui.zhang@alumni.ubc.ca (T.R.Z.); aydn1831@gmail.com (A.K.); alyssa.ash@alumni.ubc.ca (A.M.A.); 2 Non-Invasive Neurostimulation Therapies Laboratory, Department of Psychiatry, University of British Columbia, Vancouver, BC V6T 1Z3, Canada; fidel.vilarodriguez@ubc.ca; 3 Djavad Mowafaghian Centre for Brain Health, University of British Columbia, Vancouver, BC V6T 1Z3, Canada; 4 Department of Physiotherapy, Federal University of Sao Carlos, Sao Carlo 13565-905, SP, Brazil; evelynmguilherme@gmail.com

**Keywords:** adult neurogenesis, plasticity, cell proliferation, electroconvulsive shock, transcranial magnetic stimulation, theta burst

## Abstract

Hippocampal plasticity is hypothesized to play a role in the etiopathogenesis of depression and the antidepressant effect of medications. One form of plasticity that is unique to the hippocampus and is involved in depression-related behaviors in animal models is adult neurogenesis. While chronic electroconvulsive shock (ECS) strongly promotes neurogenesis, less is known about its acute effects and little is known about the neurogenic effects of other forms of stimulation therapy, such as repetitive transcranial magnetic stimulation (rTMS). Here, we investigated the time course of acute ECS and rTMS effects on markers of cell proliferation and neurogenesis in the adult hippocampus. Mice were subjected to a single session of ECS, 10 Hz rTMS (10–rTMS), or intermittent theta burst stimulation (iTBS). Mice in both TMS groups were injected with BrdU 2 days before stimulation to label immature cells. One, 3, or 7 days later, hippocampi were collected and immunostained for BrdU + cells, actively proliferating PCNA + cells, and immature DCX + neurons. Following ECS, mice displayed a transient increase in cell proliferation at 3 days post-stimulation. At 7 days post–stimulation there was an elevation in the number of proliferating neuronal precursor cells (PCNA + DCX +), specifically in the ventral hippocampus. iTBS and rTMS did not alter the number of BrdU + cells, proliferating cells, or immature neurons at any of the post-stimulation time points. Our results suggest that neurostimulation treatments exert different effects on hippocampal neurogenesis, where ECS may have greater neurogenic potential than iTBS and 10–rTMS.

## 1. Introduction

Adult hippocampal neurogenesis provides a source of cellular and synaptic plasticity that may be relevant for treating a number of psychiatric disorders [1]. A considerable amount of this work has been performed in the context of depression since there is hippocampal atrophy in patients [2], reduced neurogenesis in preclinical animal models [3,4], and manipulations that impair or enhance neurogenesis lead to greater or reduced levels of depression-like behavior in mice, respectively [5,6,7]. Given these relationships between neurogenesis and the depressive phenotype, a fundamental question is how neurogenesis can be enhanced to treat depression. While environmental enrichment [8], exercise [9], and antidepressant drugs [10] promote neurogenesis, many patients are treatment resistant and need alternative therapies [11].

Neurostimulation therapies have been used clinically for many years and continue to evolve at a rapid pace. Moreover, there are varying degrees of evidence that different neurostimulation therapies modulate hippocampal function, including neurogenesis. For example, electroconvulsive therapy (ECT) is a non-invasive treatment that triggers a controlled tonic–clonic generalized seizure using a brief electrical current under general anesthesia. It is the most efficacious treatment for severe depression and is also indicated for schizophrenia, catatonia, and active suicidal ideation [12,13,14,15]. Electroconvulsive shock (ECS), which is the ECT analog procedure in preclinical studies, has one of the most robust neurogenic effects in animal models, where both acute and chronic ECS typically elevates neurogenesis by 2–3-fold [16,17]. Furthermore, the antidepressant-like effects of ECS depend on intact hippocampal neurogenesis [18].

In contrast, transcranial magnetic stimulation (TMS) is a newer form of neurostimulation that depolarizes neurons via a changing magnetic field. TMS does not induce seizures or require anesthesia, is better tolerated than ECT [19] and is therefore a first-line treatment for (pharmacological) treatment-resistant depression [20,21]. The most commonly used form, high frequency repetitive TMS (rTMS, ~10–20 Hz) enhances hippocampal learning and induces several forms of hippocampal plasticity including long-term potentiation and the expression of BDNF and synaptic proteins [22,23,24]. rTMS also reduces depression-like behavior in animal models [25]. While rTMS has been found to increase neurogenesis in a rodent depression model [26], its effects in healthy animals are less clear, with one study reporting that rTMS elevates neurogenesis [27] and another reporting no effect [28].

Another form of rTMS, intermittent theta-burst stimulation (iTBS), delivers bursts of stimuli according to a theta pattern (~5 Hz) [29,30]. iTBS can be administered over a significantly shorter window of time than traditional, high frequency rTMS [20] and is believed to leverage the brain’s natural propensity to undergo plasticity in response to theta-patterned stimuli [31,32], which are prominent in the hippocampus [33,34,35,36]. In rats, adult-born neurons may contribute to the endogenous theta rhythm [37]. Also, theta-patterned stimulation of the perforant path, which directly innervates adult-born hippocampal neurons, reduces immobility in the forced swim test [38]. However, no studies have explored whether iTBS, such as other forms of neurostimulation, alters the production of newborn hippocampal neurons.

Neurogenesis is a multistep process involving the proliferation of neural precursors and the survival of post-mitotic neurons through multiple distinct critical periods. Identifying the net neurogenic effect of any manipulation therefore requires an assessment of its impact on each of these stages of neuron production. A number of studies have found that ECS dramatically increases cell proliferation [10,16,17] and that these additional newborn cells survive for the long-term [16,39]. However, little is known about timing of ECS effects since most studies have examined proliferation after chronic stimulation regimens. Several studies have found that a single ECS session increases proliferation 3–4 days later [40,41], raising the question of whether ECS effects are restricted to a specific window of time. There are claims that the proliferative effects of ECS are delayed, peak at 3 days, and then subside [16]. However, since these data were not shown, the time course of the proliferative effect remains unclear. Finally, while there is evidence that chronic rTMS promotes neurogenesis [28], it is unknown whether a single session of rTMS or iTBS is capable of increasing neurogenesis.

To address these gaps, here, we systematically examined how acute ECS, rTMS, and iTBS affect adult neurogenesis in the dorsal and ventral hippocampus. We focused on cell proliferation and the population of immature neurons since these developmental stages have been virtually uncharacterized with respect to acute ECS and have never been investigated in rTMS or iTBS models. For rTMS and iTBS, we also examined BrdU-labeled cells that were largely born shortly before stimulation. Our results provide the first side-by-side comparison of the neurogenic efficacy of these neurostimulation methods and indicate that, whereas ECS transiently promoted cell proliferation, iTBS and rTMS did not significantly alter any measures of adult neurogenesis.

## 2. Materials and Methods

### 2.1. Animals and General Experimental Design

**Figure 1:**
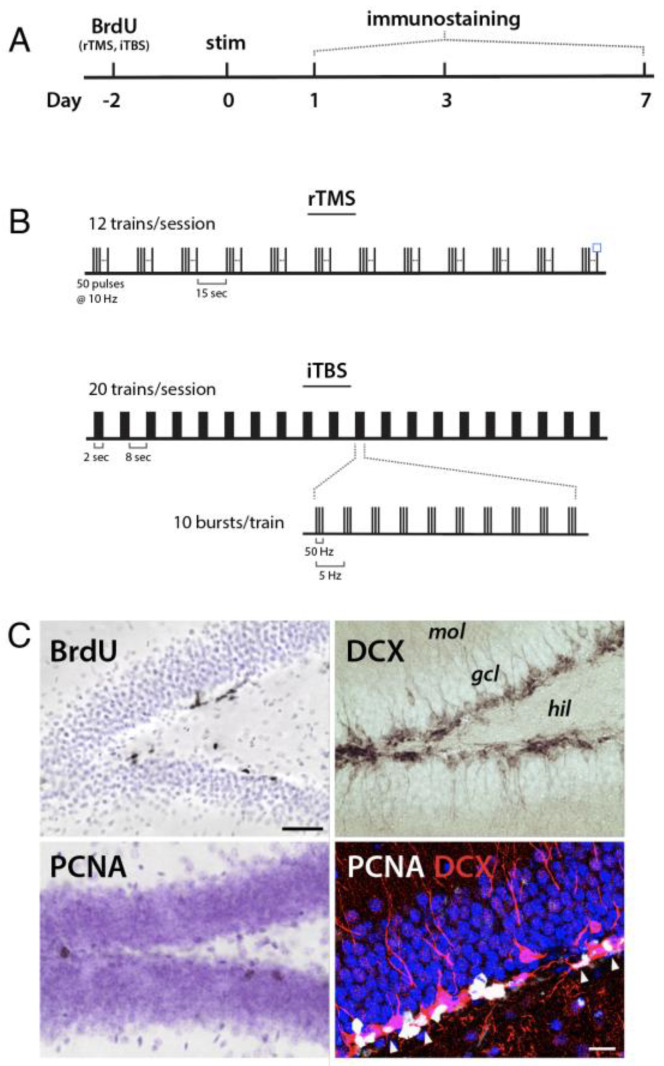
Experimental design. (**A**) Timeline of the experiment. Mice were injected with BrdU (rTMS and iTBS animals) to birthdate neurons born 2 days prior to stimulation. On day 0 mice received a single session of sham stimulation, ECS, rTMS or iTBS. Separate groups of animals were euthanized 1, 3, or 7 days after stimulation for histological analyses of neurogenic markers. (**B**) rTMS and iTBS stimulation paradigms. The smallest bar represents a single pulse at 115% motor threshold. (**C**) Representative brightfield images of BrdU, PCNA, and DCX immunostaining (scale bar, 50 µm) and confocal image of PCNA + DCX immunostaining with DAPI counterstain (scale bar, 20 µm). Arrowheads indicate PCNA + DCX + double positive cells.

All procedures were approved by the Animal Care Committee at the University of British Columbia and conducted in accordance with the Canadian Council on Animal Care guidelines. Wild-type C57BL/6 mice were bred inhouse at the Centre for Disease Modeling at the University of British Columbia. Both male and female mice were used; ECS experiments included 21 males and 26 females; TMS experiments included 22 males and 24 females. All mice were 10 to 12 weeks of age at the time of stimulation. Mice had ad libitum access to food and water and were housed on a 12 h light/dark cycle with lights on at 7 am. Animals were handled during the week before stimulation and either underwent three days of ear clip habituation (ECS animals) or one week of restraint and coil placement (TMS animals). Mice were given one stimulation session and brains were collected for histological analyses of neurogenesis at 1, 3, or 7 days following stimulation (Figure 1A). To birthdate newborn cells in the rTMS/iTBS experiment, one injection of the thymidine analog, bromodeoxyuridine (BrdU, 200 mg/kg; Toronto Research Chemicals, North York, ON, CA), was administered intraperitoneally 2 days prior to stimulation. For all experiments, equal numbers of sham animals were euthanized on days 1, 3, and 7. Since there were fewer sham animals in the ECS experiment (*n* = 2–4/timepoint) and we did not expect or observe any changes in numbers of proliferating cells or immature neurons across days in the absence of stimulation, we pooled all the sham animals from the various timepoints into a single group for comparison with the ECS groups.

### 2.2. Electroconvulsive Shock

Eleven to 15-week old mice were arbitrarily assigned to ECS and sham groups, with the exception that male and female mice were equally distributed across groups. Bilateral auricular ECS was administered via padded ear clip electrodes connected to a pulse generator (ECT Unit; Ugo Basile, Gemonio, Italy). Ear clip electrodes were soaked in saline solution and placed on the base of the pinna. Stimulation parameters were adapted from Yanpellewar et al. [41] and set at 0.5 ms pulse width, 50 Hz frequency, 0.5 s total shock duration, and 20–24 mA current. ECS stimulation generated a seizure scoring 5 on the Racine seizure scale for rodents [42] for all stimulated animals. Animals displayed a loss of posture, rearing, and tonic–clonic limb movements, with full recovery. Sham animals were handled and ear clipped in the same fashion (for 1 min) but did not receive any stimulation. Stimulated animals were removed and placed back to the home cage once they regained posture and motor function.

### 2.3. Transcranial Magnetic Stimulation

Mice were 11 to 12 weeks of age at the time of stimulation. Animals were restrained to prevent movement and were stimulated with a MagPro X100 device and a TMS coil adapted for rodents (Cool-40 coil; both from Mag Venture, Farum, Denmark). The average motor threshold was determined with a separate group of animals, before the main experiment, by stimulation with increasing power until a hindlimb motor response was evoked. Experimental stimulation power was then defined as 115% of the motor threshold.

rTMS was delivered at 10 Hz, with a 5 sec train duration and a 15 s intertrain interval over 12 trains, for a total of 600 pulses (Figure 1B). The total duration of rTMS was 3 min and 40 s. For iTBS, triplet 50 Hz bursts were repeated at 5 Hz, with a 2 s train duration and an 8 s inter–train interval over 20 trains, for a total of 600 pulses. The total duration of iTBS was 3 min and 9 s (Figure 1C). Sham-stimulated mice were restrained and treated identically with the exception that the stimulation intensity was set to 0%.

### 2.4. Tissue Preparation and Immunohistochemistry 

Mice were transcardially perfused with 4% paraformaldehyde in phosphate-buffered saline (PBS, pH 7.4). Brains remained in paraformaldehyde for 48 h before sectioning. A vibratome (Leica VT1000S, Concord, ON, CA) was used to section brains into 40 µm thick coronal sections. Brain sections were stored in cryoprotectant at −20 °C until immunohistochemical processing. BrdU was used to investigate effects of rTMS/iTBS on the survival of cells born prior to stimulation, as in [43]. Proliferation cell nuclear antigen (PCNA) is a protein involved in cell mitosis and was used as a marker for cell proliferation at the time animals were euthanized [43,44]. Doublecortin (DCX) is a microtubule-associated protein continuously expressed in neurons undergoing maturation and used here as a marker for immature neurons [43,44]. Briefly, brain slices were mounted on slides (Fisher) and allowed to dry; they were rehydrated in PBS on the day of staining. Tissue was heated in citric acid (0.1 M, pH 6.0, 20 min) for antigen retrieval. For BrdU staining, tissues were incubated in HCl following citric acid. For PCNA staining, tissues were incubated in trypsin buffer following citric acid. The tissues were then incubated in one of the primary antibodies (1:200 mouse monoclonal anti-BrdU antibody (BD Biosciences, Franklin Lakes, NJ, USA), 1:200 mouse monoclonal anti-PCNA antibody (Santa Cruz, Dallas, TX, USA), 1:250 rabbit anti-doublecortin antibody (NEB) for up to 2 days. The slides were then washed and stained with their respective secondary antibody (1:200 biotinylated goat anti-mouse antibody (Sigma Aldrich, St. Louis, MO, USA), 1:200 biotinylated donkey anti-rabbit antibody (Cedarlane, Burlington, ON, CA)). Immunopositive cells were visualized with an avidin–biotin–horseradish peroxidase kit (Vector Laboratories, Burlingame, CA, USA) and cobalt-enhanced DAB (Sigma). To identify whether proliferating cells are neuronal precursors, a double immunofluorescent immunostaining of PCNA and DCX was conducted. Slices were stained free floating in well plates. Slices were washed 3 times with PBS then heated to 95 °C in citric acid for 10 min. Slices were incubated in 1:200 mouse anti-mouse PCNA antibody (Santa Cruz) and 1:400 rabbit anti-doublecortin antibody (NEB) for 3 days. The slices were then washed and incubated with Alexa 488 donkey anti-mouse secondary antibody (Fisher, Waltham, MA, USA) and Alexa 647 donkey anti-rabbit antibody (Fisher) for 1 h. Slices were finally incubated with 1:1000 DAPI to visualize cell nuclei, mounted onto slides, and coated with anti-fading reagent PVA-DABCO. 

### 2.5. Quantification and Statistical Analysis

Positively labeled cells in the granule cell layer and the sub granular zone were manually counted on a light microscope with a 40x objective. Four hippocampal sections from each animal were chosen (two dorsal i.e., bregma −1.46 to −2.3 and two ventral i.e., bregma −3.02 to 3.6), and all positive cells were counted. Certain tissue was exhausted during the course of the experiment, and thus not all animals were examined for all neurogenesis markers. Tissue volume was obtained by tracing the 2–dimensional area of the granule cell layer and multiplying by the tissue thickness, and cell densities (in mm^3^) were then obtained by dividing cell counts by the tissue volume that was sampled.

For PCNA/DCX double labeling, images of slices were acquired with Leica SP8 confocal microscope. Images were acquired with a 40× oil immersion lens at 1.0 zoom. Images of 1024 × 1024 pixels (400 Hz scan speed) in size at a z–resolution of 1.5 µm were merged to encompass the entire dentate gyrus. Dorsal and ventral dentate gyrus sections were counted in Image J (with the Cell Counter plugin) for cells positive for PCNA and cells positive for both PCNA and DCX. The percentage of neuronal precursors was defined as the percentage of PCNA positive cells that were also DCX positive. The average percentage of neuronal precursors of each stimulation group was obtained and used to extrapolate the density of neuronal precursors.

Statistical analysis was performed using Prism 8 (Graphpad, San Diego, CA, USA), R, and SPSS. Cell counts in the ECS and rTMS/iTBS experiments were analyzed by two-way (dorsoventral subregion x treatment group) repeated measures ANOVA. In cases where tissue was not available for both subregions of a given animal, data were analyzed by mixed effects analysis. Where animal ages were not evenly distributed across groups, data were analyzed by ANCOVA, with age as a covariate. Where significant effects were observed, group differences were examined further by post hoc Tukey HSD tests. The statistical significance threshold was set at α = 0.05.

## 3. Results

### 3.1. Effects of Acute ECS on Dentate Gyrus Neurogenesis

**Figure 2:**
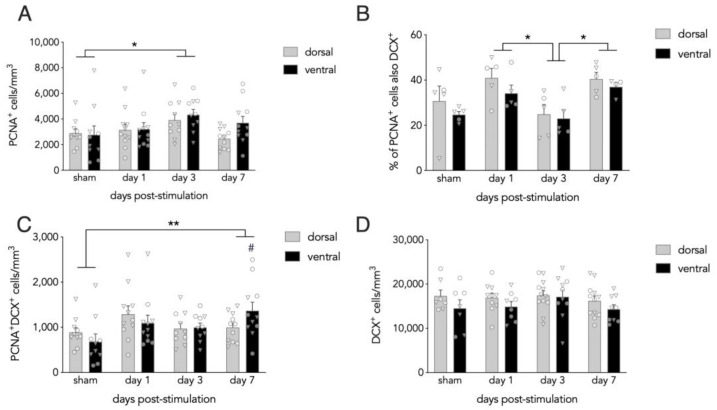
Time course of neurogenesis following one session of ECS. (**A**) The density of proliferating PCNA + cells increased on day 3 post-ECS compared to sham animals and was similar in the dorsal and ventral dentate gyrus. (**B**) The proportion of PCNA + cells that expressed DCX was lower on day 3 than on days 1 and 7. (**C**) The total number of PCNA + DCX + cells was elevated on day 7 relative to the sham group and elevated in the ventral DG relative to all other groups. (**D**) ECS did not alter DCX + cell density. Bars reflect mean ± standard error. Triangles indicate female mice; circles indicate male mice. * *p* < 0.05, ** *p* < 0.01, ^#^ *p* ≤ 0.001/0.03/0.02 vs sham/day 1/day 3.

To determine whether acute ECS elevates cell proliferation at specific timepoints, we quantified PCNA+ cells at 1, 3, and 7 days post-stimulation and analyzed by repeated measures ANCOVA (treatment × dorsoventral subregion), with age as a covariate since animal ages were not evenly distributed across experimental groups. PCNA+ cell densities progressively increased from days 1 to 3 post-ECS and were ~60% greater than shams at day 3. By day 7, the density of PCNA+ cells declined and was comparable to sham levels. PCNA cell densities did not differ along the dorsoventral axis (group effect: F_3,37_ = 3.1, *p* = 0.037; dorsoventral effect: F_1,37_ = 2.6, *p* = 0.11; dorsoventral interactions all *p* > 0.8; sham vs. day 3: *p* = 0.015, all other comparisons *p* > 0.17; Figure 2A).

The PCNA+ cell population is heterogeneous and composed of multiple types of dividing cells. To determine whether ECS specifically alters the proliferation of neuronal precursors, we quantified the proportion of PCNA + cells that also expressed the immature neuronal marker DCX. Here, we found that 20–40% of PCNA + cells also expressed DCX, with more cells expressing DCX on days 1 and 7 compared to day 3 (mixed effects analysis: F_3,16_ = 5.4, *p* = 0.009; day 1 vs 3: *p* = 0.03; day 7 vs 3: *p* = 0.03; all other comparisons *p* > 0.12; Figure 2B). We next multiplied the PCNA cell counts by the % DCX expression to estimate changes to the total number of neuronal precursors and analyzed the data by repeated measures ANCOVA with animal age as a covariate. This revealed a main effect of group and a significant group × subregion interaction (group effect: F_3,37_ = 5.1, *p* = 0.005; subregion effect: F_1,37_ = 1.4, *p* = 0.24; group × subregion interaction: F_3,37_ = 4.0, *p* = 0.014). Post hoc analyses revealed greater numbers of double positive PCNA + DCX + cells on day 7 compared to shams (sham vs ECS days 1/3/7: *p* = 0.2/0.8/0.004), an effect that was specific to the ventral DG (dorsal: all comparisons *p* ≥ 0.2; ventral: day 7 vs sham/day 1/day 3 *p* = < 0.001/0.03/0.02; all other comparisons *p* > 0.37).

PCNA labels cohorts of cells that are born at discrete points in time and therefore cannot reveal how acute stimulation may impact broader populations of immature neurons. To determine how a single ECS session impacts the general population of newborn neurons, we quantified DCX + cells, which reflects immature neurons up to ~3–4 weeks of age [43]. We found no differences between sham and ECS-treated groups (mixed effects analysis; effect of group: F_3,37_ = 1.0, *p* = 0.4; effect of subregion: F_1,30_ = 3.8, *p* = 0.056; group x subregion interaction: F_3,30_ = 0.3, *p* = 0.8; Figure 2D).

### 3.2. Effects of Acute rTMS and iTBS on Dentate Gyrus Neurogenesis

**Figure 3:**
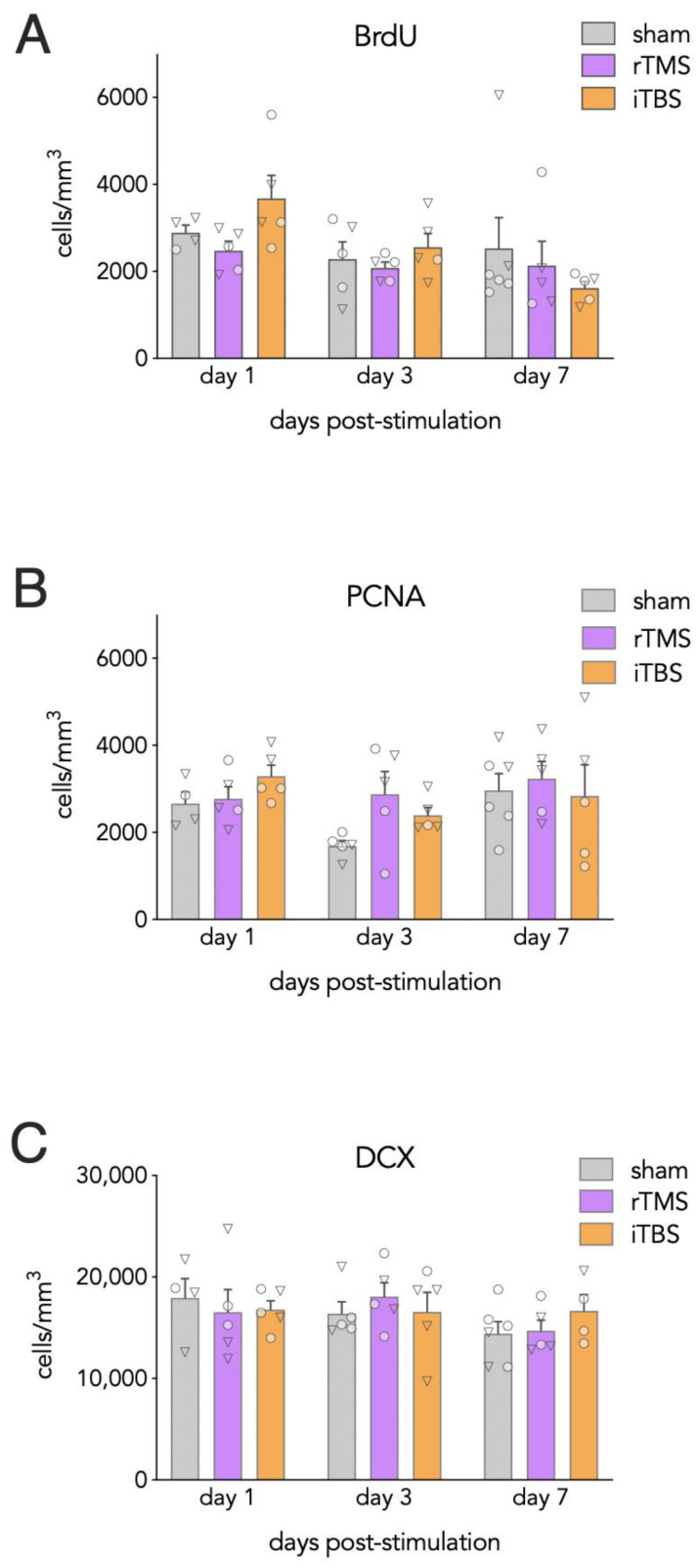
Time course of neurogenesis following one session of rTMS and iTBS. (**A**) Neither stimulation treatment altered the survival of BrdU + cells relative to shams. (**B**) The density of proliferating PCNA + cells was not altered by rTMS or iTBS treatments. (**C**) The density of proliferating immature DCX + cells was not altered by rTMS or iTBS treatments. Bars reflect mean ± standard error. Triangles indicate female mice; circles indicate male mice.

Stimulated mice were examined at 1, 3, or 7 days post-stimulation and compared to sham-stimulated mice. Stimulation did not differentially modulate markers of neurogenesis across the dorsoventral axis (Supplementary Materials, Figure S1) and thus all cell marker data were pooled for subsequent analyses. The density of BrdU + cells declined over days but did not vary as a function of type of TMS (ANOVA; effect of day: F_2,36_ = 3.6, *p* = 0.039; effect of group: F_2,36_ = 0.70, *p* = 0.50; Figure 3A). While there was no significant group × day interaction (F_4,36_ = 1.5, *p* = 0.2), exploratory analyses suggest that the decline in BrdU + cells over days appeared to be driven by the iTBS group, which had more than twice as many BrdU + cells on day 1 compared to day 7 (*p* = 0.046; sham and rTMS: day 1 vs day 7, both *p* > 0.9).

PCNA + cell densities were analyzed to determine rTMS and iTBS–related changes in cell proliferation. Here, no differences were observed across groups (effect of group: F_2,39_ = 1.4, *p* = 0.3; day × group interaction: F_4,39_ = 0.9, *p* = 0.5; Figure 3B).

DCX + cell densities were analyzed to assess stimulation-related changes in immature neuron numbers. However, no differences were observed between sham and stimulated mice groups (effect of group: F_2,35_ = 0.06, *p* = 0.9; day × group interaction: F_4,35_ = 0.6, *p* = 0.7; Figure 3C).

## 4. Discussion

Here, we examined the effects of a single ECS, iTBS, or rTMS session on markers of proliferation and neurogenesis in the adult mouse hippocampus. By using immunohistochemical markers for birthdated BrdU + cells, proliferating cells, and immature neurons, we were able to investigate several neurogenic cell populations in the same animals. ECS had the greatest effects, with increased cellular proliferation at 3 days and neuronal precursor proliferation at 7 days post-stimulation. In contrast neither iTBS nor rTMS altered numbers of proliferating cells or immature DCX + cells. In our TMS experiment, we also examined whether stimulation alters the number of BrdU + cells (largely born shortly before stimulation). Here, we found that both iTBS and rTMS did not alter BrdU + cells, though our exploratory analyses suggest that recently born neurons may be culled at a faster rate following iTBS. Collectively, these findings provide new insights into the temporal specificity and efficacy by which different neurostimulation modalities impact neurogenesis.

### 4.1. ECS and Neurogenesis

ECS is arguably one of the most robust neurogenic stimuli identified to date and typically elevates neurogenesis by 2–3-fold compared to baseline levels [16,45,46]. Naturally, since ECS models ECT and ECT is administered in a chronic fashion, most animal studies have employed chronic ECS paradigms in which BrdU is administered throughout the stimulation window to assess neurogenesis levels [39,47,48,49]. While this approach may maximize new neuron labeling, it cannot distinguish whether ECS alters neurogenesis at different stages of the stimulation paradigm. Similarly, while BrdU given at the end of a chronic stimulation indicates that ECS promotes the proliferation of neural precursor cells [48,50]. This design cannot pinpoint precisely when, during the stimulation paradigm, enhanced proliferation begins.

Here, we used a single stimulus to refine the exact point at which ECS promotes proliferation in mice. Previous studies in rats have reported that a single ECS treatment can promote proliferation 2–3 days later [40,41,46,51]. Whereas these studies did not examine other timepoints, Madsen et al. [16] claimed that the proliferative effects of ECS are transient and peak at 3 days. However, these data were not provided in their study. Our findings therefore confirm that a single ECS treatment transiently elevates proliferation 3 days later, and they also indicate that this occurs in mice in addition to rats. While proliferation was similarly elevated in the dorsal and ventral hippocampus, we observed greater numbers of proliferating neuronal precursor cells (PCNA + DCX +), specifically in the ventral dentate gyrus. This aligns with previous evidence that neurogenesis in the ventral dentate gyrus is particularly sensitive to antidepressant treatment [52].

In contrast to its effects on proliferation, ECS did not significantly alter the density of immature DCX + cells during the week, following stimulation. This may be because the transient rise in proliferation produced a relatively small increase in new cells compared to the large number of immature neurons that express DCX. Alternatively, we did observe an increase in the density of proliferating neuronal precursors at 7 days post-ECS and thus it is possible that there is a delayed increase in neuron production due to the time required for seizures to promote the division of radial stem cells and birth of the transit amplifying cells that are ultimately responsible for producing newborn neurons [53]. Future experiments may therefore examine whether acute ECS can increase DCX+ cell number beyond the 1 week interval examined here. Nevertheless, since chronic ECS consistently increases numbers of DCX + cells [17,41,48], it appears that, with additional stimulation, ECS results in a net gain of newborn neurons rTMS, iTBS and neurogenesis.

Whereas ECS directly depolarizes large populations of neurons through applied current and leads to generalized seizures, TMS induces milder degree of neuronal depolarization via a changing magnetic field and does not trigger seizures [54]. While the mechanisms of TMS action are not entirely clear, high frequency rTMS (>5 Hz) tends to excite neural circuits and lower frequency stimulation (≤1 Hz) tends to have inhibitory effects [55]. Inspired by animal work showing that long-term potentiation can be reliably induced by theta-patterned stimulation [31,32], theta-related TMS protocols were developed, of which iTBS was found to be particularly effective at potentiating motor pathways in humans [30]. Since the theta rhythm is intimately linked to hippocampal function [56], we anticipated that iTBS may be particularly capable of promoting adult hippocampal neurogenesis. Indeed, previous studies have found that newborn neurons undergo robust long-term potentiation in response to theta burst direct electrical stimulation of the perforant path [57,58], and blocking neurogenesis is associated with reduced hippocampal theta power and impaired associative learning [37].

By all measures, TMS had weaker effects on neurogenesis measures than ECS. Whereas chronic rTMS has been found to increase neurogenesis in several studies [26,27], a single treatment did not alter the number of proliferating, surviving, or immature neurons. However, our exploratory analyses suggest that iTBS may reduce survival of immature BrdU + cells across days 1–7. Since we did not observe any increase in proliferation (as in the ECS experiment), it appears that any iTBS-induced cell loss occurs independent of cell addition. However, another possibility is that our experimental design failed to capture a transient proliferative effect of iTBS, or that iTBS induced other forms of activity or plasticity that led to a compensatory loss of immature cells. Given that chronic stimulation may exert a more reliable neurogenic effect, and physiological effects of TMS depend on the number, frequency, and pattern of stimuli [55], future experiments are needed to clarify whether other TMS stimulation paradigms are capable of promoting neurogenesis.

### 4.2. Parallels with Clinical Findings

Our experimental design intended to maximize similitudes with clinical applications of neurostimulation treatments in humans in an effort to increase the translational interpretation of our results (e.g., rTMS protocol is similar to the 10Hz protocol in humans, and iTBS protocol is identical to the protocol used in humans). Interest in neurogenic treatments stems from findings that hippocampal dysfunction, including structural atrophy and volume loss, is observed in multiple disorders such as depression [2], schizophrenia [59], and Alzheimer’s disease [60]. Neurogenesis is often cited as a possible mechanism for restoring hippocampal function and behavioral outcomes, and therefore, significant efforts have been devoted in identifying methods for promoting the birth and survival of newborn neurons. Arguably, neurogenesis is most consistently associated with depression. In animals, adult-born neurons regulate both the behavioral and HPA response to stress [61,62,63], and they have been found to reduce depression- and anxiety-related behaviors in multiple tasks [5,64,65]. Numerous stimuli that have antidepressant properties are known to increase neurogenesis, including exercise [9], antidepressant drugs [10], ECS [10,16], and TMS [27]. Neurogenesis is required for certain behavioral effects of both SSRI antidepressant drugs [62] and ECS [18] in mouse models. While the biological basis of depression extends beyond neurogenesis, and new neuron production is unlikely to fully account for changes in hippocampal volume [66], recent human work suggests that the hippocampus is a relevant therapeutic target for stimulation therapies. ECT reliably increases hippocampal volume [67,68,69,70]. Furthermore, hippocampal subregional analyses have found that ECT specifically increases dentate gyrus volume [71] and the magnitude of the volume increase correlates with clinical improvement [72,73,74]. Depression is associated with fewer dentate gyrus granule cells [75,76], but cell numbers can be restored by antidepressant drug treatment [77], suggesting that either increased production of new cells or reduced death of old cells [44] may be involved. 

While there are debates regarding the magnitude of adult hippocampal neurogenesis [78,79,80,81], it is likely that, even in the absence of high rates of neurogenesis, prolonged dentate gyrus development contributes to extended hippocampal plasticity in humans [82]. For example, we recently reported that adult–born neurons mature over ~25% of the rodent lifespan. Coupled with low rates of ongoing neurogenesis, the cumulative plasticity afforded by later-born neurons lasts until the end of life [83]. Since it is known that adult-born neuron physiology and connectivity is highly sensitive to experience [84,85,86], stimulation therapies can reasonably be expected to impact hippocampal circuits by altering excitability, plasticity, or wiring of new neurons in the absence of effects on cell proliferation or survival. 

### 4.3. Limitations

There are several limitations of this study that are important to consider. First, compared to clinical settings, neurostimulation treatments have low spatial resolution in mouse models. Whereas TMS typically targets the dorsolateral prefrontal cortex in humans, modeling work in rodents estimates that our TMS coil generates a magnetic field that likely directly stimulates the entire mouse brain [87], including deeper structures such as the hippocampus. It is therefore unclear, for example, whether iTBS effects on BrdU + cell density would be observed if stimulation was restricted to frontal neocortical regions. However, recent work shows that TMS applied to the parietal lobe can promote hippocampal activity and memory function through indirect effects on connected circuits [88]. Thus, TMS effects on hippocampal function in mice may serve as a reasonable model for certain forms of TMS applied to humans.

Second, due to tissue limitations, we relied on cell density measurements rather than stereological cell quantification. This may be an issue if treatments induced changes in tissue volume. However, ECT increases hippocampal volume in patients [70], which will only serve to underestimate cell counts and, therefore, cannot explain the increase in cell proliferation that we observed on day 3. While volume increases can theoretically underlie the loss of BrdU + cells from days 1–7 in iTBS mice, this seems unlikely given that a previous study found that nine sessions of ECS did not alter hippocampal volume in rodents [49].

Finally, we injected BrdU 2 days before rTMS and iTBS to monitor survival of immature cells. While this will primarily label cells born before stimulation, it is possible that a small number of label-retaining precursor cells may have been dividing at the time of stimulation [89], explaining why BrdU is (nonsignificantly) elevated 1 day after iTBS treatment. Thus, it is possible that the loss of BrdU + cells from days 1–7 reflects a mixed population of neurons born shortly before and after stimulation.

### 4.4. Conclusion and Future Directions

In summary, among the three neurostimulation methods tested, neurogenic effects are robust following acute ECS, and weak or absent following acute iTBS and rTMS. Given that rTMS can promote neurogenesis in chronic settings, it will be worthwhile to investigate whether chronic iTBS also influences neurogenesis to a similar extent. Given the high degree of plasticity of newborn neurons, it will also be important for future studies to examine the functional properties of neurons that are subjected to neurostimulation and determine their role in the memory- and mood-related functions of the hippocampus.

## Data Availability

The data presented in this study are available on request from the corresponding author.

## Supplementary Materials

The following are available online at https://www.mdpi.com/article/10.3390/cells10082090/s1, Figure S1: Dorsal-Ventral comparison of neurogenesis markers following acute TMS stimulation.
