# Supplementary material for: Electroconvulsive Shock, but Not Transcranial Magnetic Stimulation, Transiently Elevates Cell Proliferation in the Adult Mouse Hippocampus
Source: Cells. 2021 Aug 14;10(8):2090. doi: 10.3390/cells10082090 (PMC8391684; doi:10.3390/cells10082090)
Supplement: Supplementary file 1 [file cells-10-02090-s001.zip › cells-1125088-supplementary.pdf]

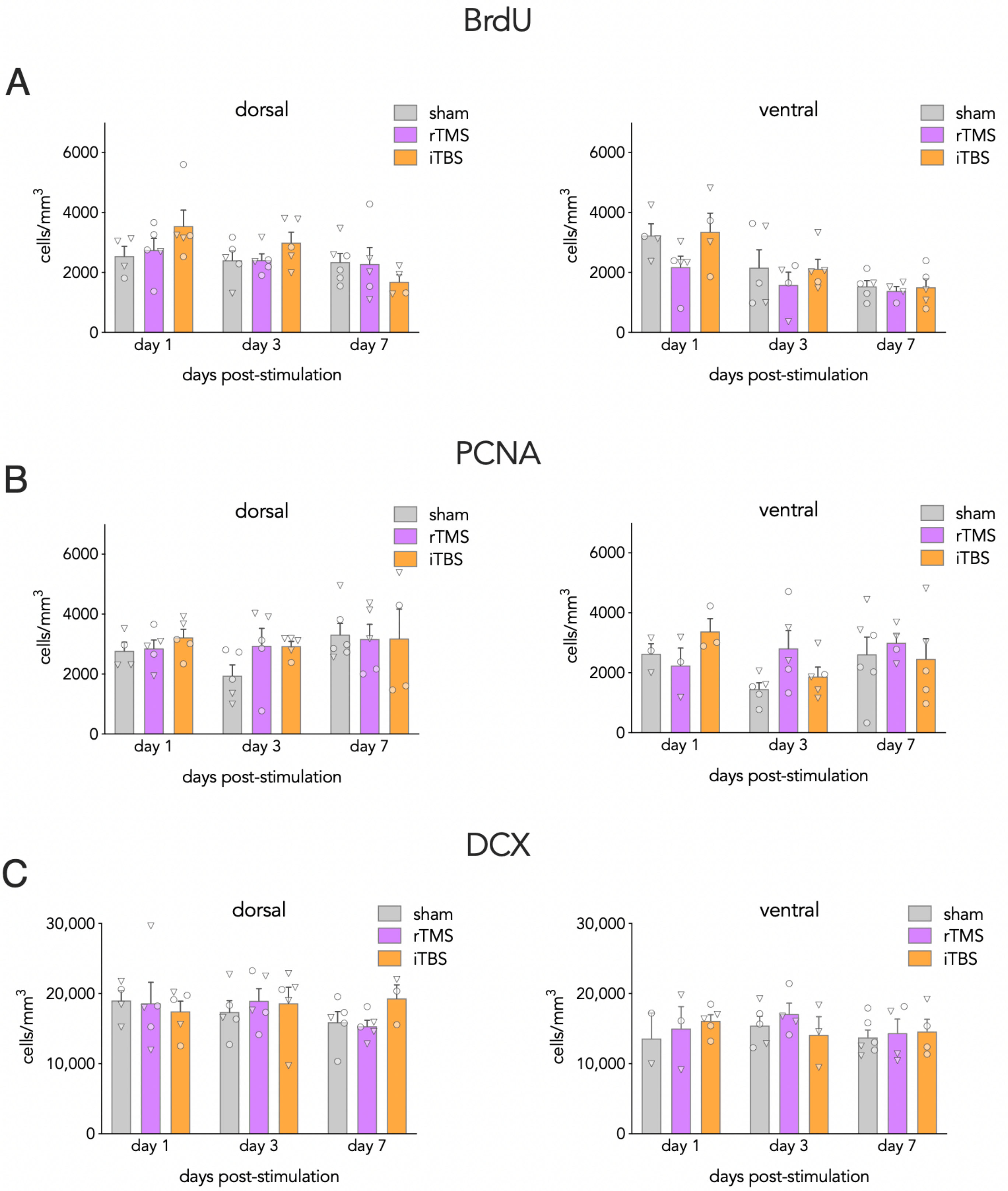
BrdU
A
dorsal
ventral
sham
rTMS
iTBS
cells/mm3
0
2000
4000
6000
day 1
day 3
day 7
days post-stimulation
PCNA
B
dorsal
ventral
sham
rTMS
iTBS
cells/mm3
0
2000
4000
6000
day 1
day 3
day 7
days post-stimulation
DCX
C
dorsal
ventral
sham
rTMS
iTBS
cells/mm3
0
10,000
20,000
30,000
day 1
day 3
day 7
days post-stimulation

Supplementary Figure 1: Timecourse of neurogenesis, in the dorsal and ventral dentate gyrus, following one session of rTMS and iTBS. A) BrdU+ cell densities did not vary as a function of dorsoventral subregion (sham vs rTMS 3 way ANOVA: dorsoventral main effect and interactions all $P > 0.11$; sham vs iTBS 3 way ANOVA: dorsoventral main effect and interactions all $P > 0.22$). B) PCNA+ cell densities did not vary as a function of dorsoventral subregion (sham vs rTMS 3 way ANOVA: dorsoventral main effect and interactions all $P > 0.10$; sham vs iTBS 3 way ANOVA: dorsoventral main effect and interactions all $P > 0.09$). C) DCX+ cell densities were greater in the dorsal dentate gyrus but dorsoventral differences were not modulated by treatment or time post-stimulation (sham vs rTMS 3 way ANOVA; dorsoventral effect: $P = 0.04$; sham vs iTBS 3 way ANOVA; dorsoventral effect: $P = 0.004$; all dorsoventral interactions $P > 0.3$). Bars reflect mean ± standard error. Triangles indicate female mice, circles indicate male mice.
